# Spinal Muscular Atrophy in Blonde D'Aquitaine Calves Is Not Associated With FVT1 Gene Mutation

**DOI:** 10.3389/fvets.2020.00348

**Published:** 2020-06-23

**Authors:** Giulia Cagnotti, Carlo Cantile, Stefania Chessa, Paola Sacchi, Antonio D'Angelo, Claudio Bellino

**Affiliations:** ^1^Department of Veterinary Science, University of Turin, Turin, Italy; ^2^Department of Veterinary Sciences, University of Pisa, Pisa, Italy

**Keywords:** cattle, neurodegenerative diseases, motor neuron diseases, spinal muscular atrophy, genetic mutation

## Abstract

Spinal muscular atrophy (SMA) is a motor neuron disease (MND) in humans and diverse animal species: canid, felid, and bovid. To date, bovine SMA has been reported in Brown Swiss, Holstein, Friesian, and Red Danish breed; it has been associated with a genetic mutation of the FVT1 gene, also known as 3-ketodihydrosphingosine reductase (KDSR). The aim of the present case series was to describe clinical presentation, pathological findings, and genetic analysis of five Blond d'Aquitaine calves diagnosed with SMA and to determine whether the mutation was associated with the disease. Five Blonde d'Aquitaine calves (three females and two males) from the same cow-calf operation farm were presented between June 2018 and February 2019 because unable to stand or walk unassisted since birth. Neurological examination aroused suspicion of a diffuse lesion affecting the peripheral nervous system in all calves. Findings from electromyographic investigations and muscle and nerve biopsies were consistent with a non-regenerative, chronic, active axonal neuropathy and marked neurogenic muscular atrophy and assumed to be associated with a neurodegenerative process. Histopathological examination of tissue samples from two animals revealed neuronal loss and several degenerated, shrunken, and hypereosinophilic neurons at the level of the ventral horn of the cervico-thoracic and the lumbo-sacral intumescence, diffuse loss of myelinated axons at the level of the ventral funiculi of all segments of the spinal cord, and moderate diffuse astrocytic reaction. These findings confirmed the diagnosis of SMA. No mutation of the FVT1 gene was found on genetic analysis. Further study into the causative gene mutation of SMA in Blonde D'Aquitaine calves is under way. Identification of a novel genetic mutation could improve our understanding of the disease in human medicine.

## Background

SMA is a MND in humans and diverse animal species: canid, felid and bovid (1–6). These central nervous system disorders usually affect somatic lower motor neurons of the anterior/ventral horns of the spinal cord and surrounding myelin; other regions of the central nervous system, including the brainstem and the motor cortex, can be affected as well. Clinically, MNDs are characterized by neurogenic muscle atrophy, paresis, and hyporeflexia usually without sensory involvement.

The etiology of these syndromes in human medicine is broadly classified as genetic, though sporadic and immune-mediated cases can also occur (7). To date, bovine SMA has been reported in Brown Swiss, Holstein, Friesian, and Red Danish breed; it has been associated with a genetic mutation of the FVT1 gene, also known as 3-ketodihydrosphingosine reductase (KDSR) (8). KDSR encodes one of several enzymes of the glycosphingolipid pathways that catalyze the second step in the biosynthesis of the sphingosine and ceramide central precursors of this pathway. A transition from G to A at the first nucleotide of codon 36 of exon 6, determining the substitution of Ala_175_ with Thr_175_, is thought to be the causative mutation of SMA or perhaps a marker in perfect linkage disequilibrium with the causative mutation (8). The aim of the present case series was to describe the clinical presentation, pathological findings, and genetic analysis of five Blond d'Aquitaine calves diagnosed with SMA and to determine whether the mutation was also associated with the disease.

## Case Description

Five Blonde d'Aquitaine calves (three females and two males) from the same cow-calf operation farm were presented to the Mobile Clinic Service of the Veterinary Teaching Hospital, Department of Veterinary Science of Turin (Italy) between June 2018 and February 2019 because they were unable to stand or walk unassisted since birth. The median age at presentation was 10 days (range 7–14); all calves were born by natural service from the same bull. Clinical examination was normal in three calves; pressure sores were noted in two; generalized muscle atrophy was also appreciable. Neurological examination performed by a board-certified neurologist (A.D.A.) was comparable for all patients. The calves were alert and responsive. Gait evaluation revealed a non-ambulatory flaccid tetraparesis with diffuse hyporeflexia in all four limbs. Cranial nerve examination revealed no abnormalities and no pain upon palpation of the vertebral coloumn and muscles. Findings from the neurological examination aroused suspicion of a diffuse lesion affecting the peripheral nervous system. Based on animals age, history and clinical presentation, a congenital or degenerative disease was suspected. Differential diagnoses included a metabolic/toxic disease or an infectious/inflammatory process.

Hematology and serum biochemistry were unremarkable, except for neutrophilic leukocytosis in two animals (8.10 × 10^3^ cells/μl and 7.55 × 10^3^ cells/μl, respectively; reference range 1.1–3.6 × 10^3^ cells/μl) attributed to inflammation of the pressure sores noted at clinical examination. Cerebrospinal fluid collected from the lumbar cistern was normal.

Electromyography performed in one animal revealed spontaneous muscular activity characterized mainly by fibrillation potentials and positive sharp waves in the appendicular and the paravertebral muscles: supraspinatus, infraspinatus, biceps, triceps, and extensor carpis radialis muscles of the thoracic limbs; quadriceps, gluteus, tibialis cranialis muscles of the pelvic limbs, and epaxial muscles. Electromyography was performed by means of Myto electromyography system (EB Neuro, Firenze, Italy) and coaxial needle electrodes for electromyography (Bionen s.a.s, Firenze, Italy).

**Figure 1:**
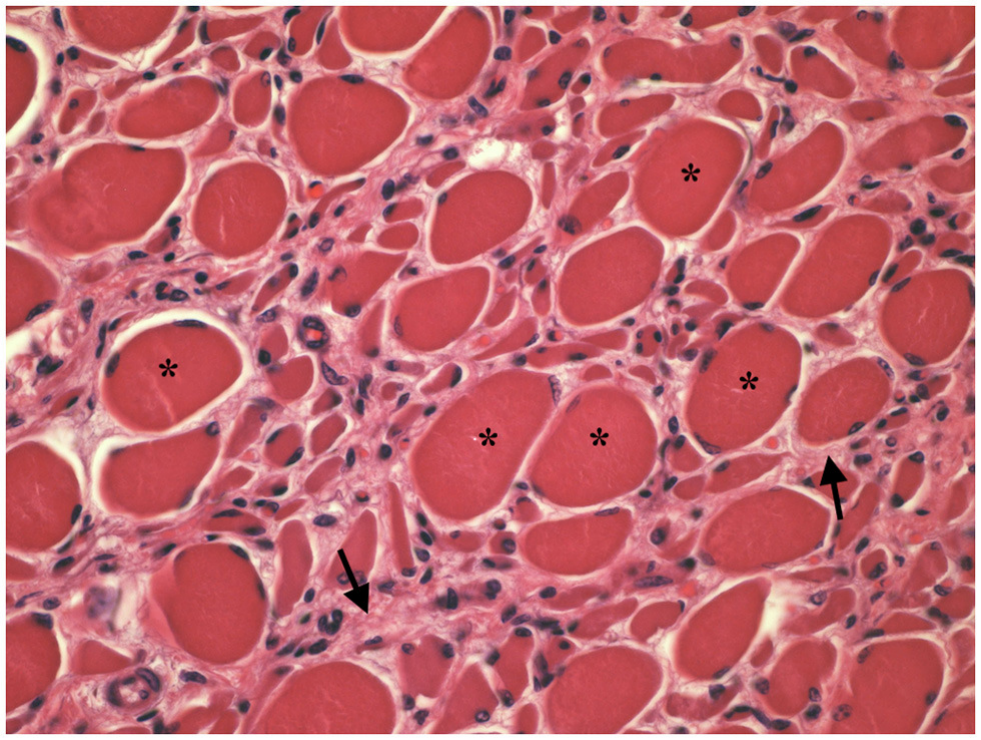
Transverse section of tibialis cranialis. Diffuse neurogenic muscular atrophy with atrophic angular fibers and hypetrophic fibers (asterisks). Endomysial fibrosis is also evident (arrows). HE × 400.

**Figure 2:**
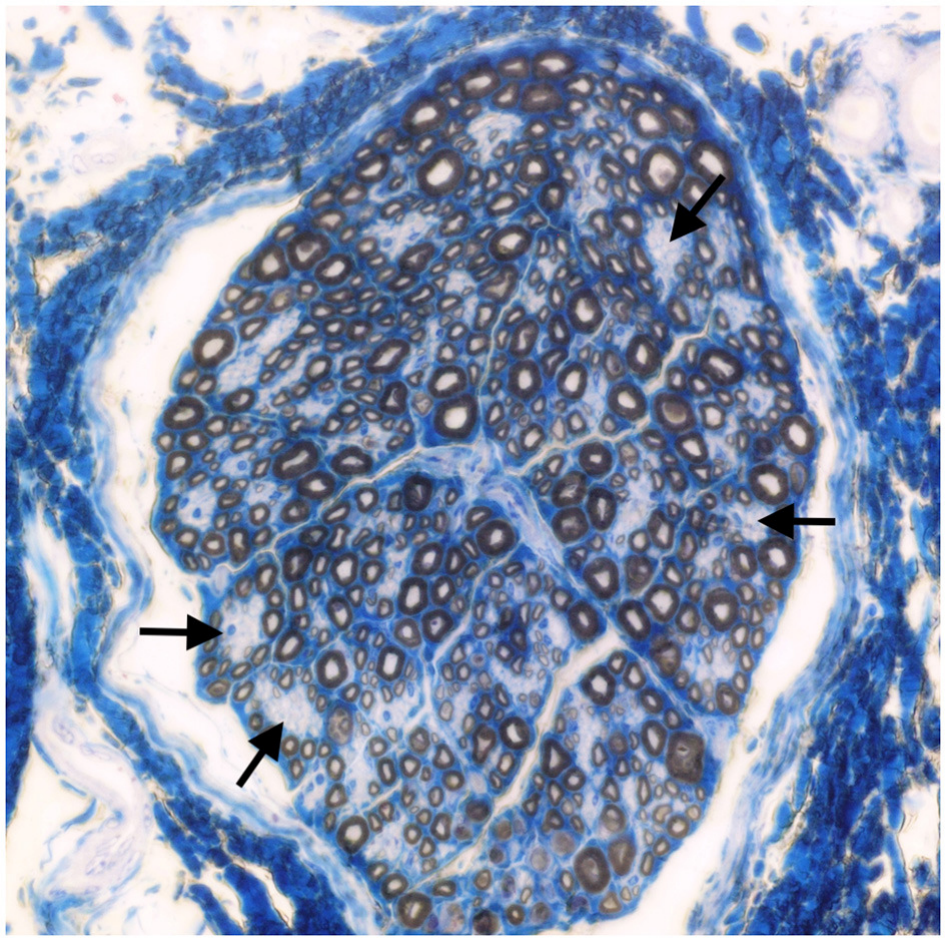
Transverse section of the peroneal nerve. Patchy loss of large and medium size myelinated fibers (arrows). Methylene blue ×500.

Muscle (triceps brachialis and tibialis cranialis) and nerve (common peroneal) biopsy from the same calf were performed on the side contralateral to electromyography. Biopsy samples were obtained by an open procedure. Transverse muscle cryosections (10-μm thick) were stained with hematoxylin-eosin, modified Gomori trichrome, Periodic-acid Schiff, Oil Red O, ATPase pH 9.8, ATPase pH 4.3, esterase, nicotinamide adenine dinucleotide, succinate dehydrogenase, and cytochrome oxidase. Nerve biopsy was fixed in 2.5% glutaraldehyde in 0.1 M cacodylate buffer at 4 °C, post fixed in OsO_4_, and embedded in Epon Araldite. Transverse semi-thin (1 μm) sections were stained with methylene blue and observed at light microscope. Histopathological evaluation of the muscle biopsies revealed marked reduction in myofiber diameter, with grouped angular and round-shaped atrophic fibers, associated with multifocal hypertrophic myofibers. Terminal stage muscle atrophy with perimysial and endomysial fibrosis and pseudo-multiplication of nuclei was noted (Figure 1). No abnormalities in myofiber distribution and oxidative pattern or lipid or glycogen sarcoplasmatic accumulation were observed. Intramuscular nervous fibers displayed loss of myelinized fibers with multifocal axonal degeneration. Examination of peripheral nerve samples revealed patchy loss of large and medium size myelinized fibers, with diffuse axonal degeneration and absence of regenerating fibers. Moderate endoneurial fibrosis was also noted (Figure 2).

**Figure 3:**
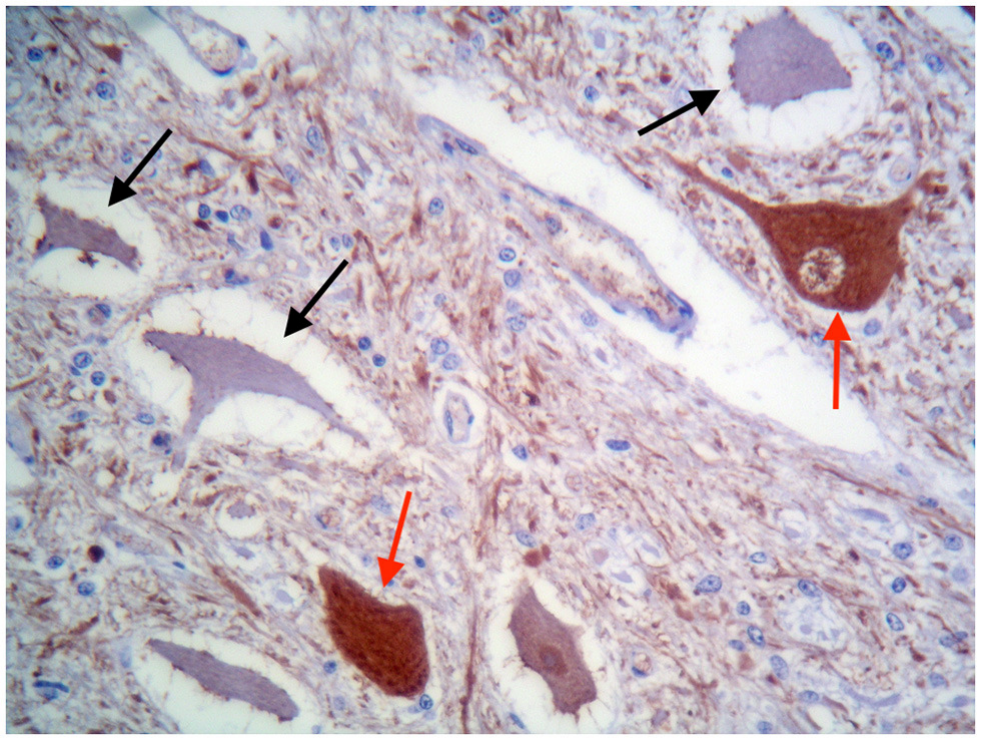
Ventral horn of the cervico-thoracic spinal cord. Degenerated shrunken motor neurons do not express NSE (black arrows), whereas morphologically normal neurons are immunolabelled (red arrows). IHC for NSE, ×400.

**Figure 4:**
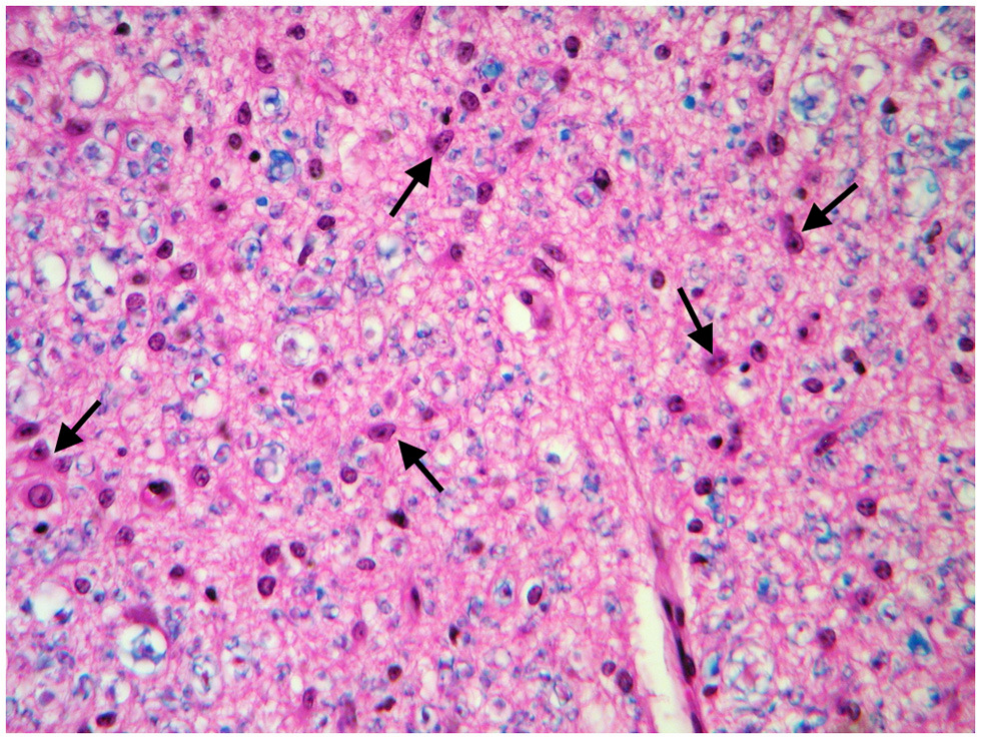
Transverse section of the spinal cord at the level of ventral funiculi. Marked loss of myelinated axons is associated with hypertrophic astrocytes (arrows). LFB-HE, ×320.

These findings were consistent with non-regenerative, chronic, active axonal neuropathy and marked neurogenic muscular atrophy assumed to be consistent with a neurodegenerative process. SMA was suspected given the young age of the animals. In light of the poor prognosis of SMA and for animal welfare reasons, all five calves were humanely euthanized and post-mortem examination was performed in two. No macroscopic changes were found, except for the pressure sores noticed in two animals upon clinical examination. Representative samples of brain and spinal cord tissues were fixed in 10% formalin solution. Transverse sections were routinely processed for histology and 4-μm sections were stained with hematoxylin-eosin and Luxol fast blue. Selected sections were stained immunohistochemically with polyclonal rabbit antibody against glial fibrillary acidic protein (GFAP 1:1000; Dako, Z0334, Carpinteria, CA, USA) and monoclonal mouse antibody against neuron-specific enolase (NSE 1:100; Dako M0873). Histopathological examination revealed neuronal loss and several degenerated, shrunken hypereosinophilic neurons at the level of the ventral horn of the cervico-thoracic and the lumbo-sacral intumescence. Immunoreactivity with anti- neuron-specific enolase antibodies was not expressed by degenerated neurons (Figure 3). Diffuse loss of myelinated axons was observed at the level of the ventral funiculi of all segments of the spinal cord (Figure 4) and immunohistochemistry with glial fibrillary acidic protein revealed moderate diffuse astrocytic reaction. No remarkable changes were observed in the brain. The histopathological findings in the tissue samples from muscle, peripheral nerve, and spinal cord were consistent with a diagnosis of SMA.

Genetic investigation was performed based on observations from a previous study (8). Genomic DNA was obtained from blood samples from the calves and the dams and the bull. Extraction was performed using the NucleoSpin® Tissue kit (Macherey-Nagel, Düren, Germany) and a region of 183 bp was amplified with the primers described in literature (8) using the HotStar Taq DNA Polymerase (Qiagen, Hilden, Germany). The PCR fragments were then sequenced by the LightRun Tube Sanger sequencing service of Eurofins genomics (Ebersberg, Germany) and visualized, aligned, and edited using BioEdit (Hall TA (1999) BioEdit: a user-friendly bio-logical sequence alignment editor and analysis program for Windows 95/98/NT. Nucl Acids Symp Ser 41:95–98). Genetic investigations did not reveal mutations affecting the FVT1 gene in any of the animals tested. To exclude other possible mutations in the same exon, a 336 bp fragment corresponding to the entire exon 6 of KDSR was also amplified using the primers Fvt_ex6_f and r (CCCTGGGCTGAAAGGAATCA and TGGGGCAGGGTACCTGAG, respectively), sequenced, and analyzed as described above. No differences in any of the samples were found, however.

## Discussion

To our knowledge, this is the first clinical, anatomopathological, and genetic description of SMA in Blonde D'Aquitaine calves.

The first consistent description of SMA in cattle was reported in 19 Brown Swiss calves in 1989. Clinical signs included initial pelvic limb weakness at 3–4 weeks of age progressing to tetraparesis and recumbency associated with severe muscle atrophy. On autopsy, the main pathological findings were motor neuron degeneration and loss in the ventral horns of the spinal cord, with accumulation of neurofilaments and mitochondria in the affected cells, and severe neurogenic atrophy (3). A few years later, Troyer and colleagues published a description of the clinical and neuropathological aspects of SMA in 53 calves of the same breed (9). A similar clinical presentation was reported in Braunvieh calves and in cross calves of the two breeds (5, 6). In 1994, SMA was described in Red Danish calves. Based on histopathology of spinal cord and muscle tissue samples from 162 Red Danish calves with suspected SMA, 82 were diagnosed with the disease. Age ranged widely between those recumbent since birth and 21-week-olds. Lesions were consistent with neuronal degeneration and denervation muscular atrophy. A familial pattern, traced back to American Brown Swiss Bulls, was identified at the time of the study (10). In 1997, Pumarola and colleagues described SMA in five Holstein-Friesian calves diagnosed on post-mortem examination. The initial clinical signs of weakness noted at the age of 15 days rapidly progressed to tetraparesis in 2 weeks. Electromyography was consistent with denervation and neuropathological examination disclosed degeneration and loss of motor neurons in the spinal cord (mainly in the ventral horn of the cervico-thoracic and the lumbo-sacral intumescence), presence of ghost cells, moderate astrocytosis, and accumulation of phosphorylated neurofilaments.

The primary pathogenic processes underlying MND are believed to be multifactorial, but the mechanism underlying selective motor neuron death remains unknown. Numerous pathogenic mechanisms contributing to motor neuron injury and cell death in MND (and neurodegenerative diseases in general) have been suggested: genetic factors, oxidative stress, excitotoxicity, protein aggregation, mitochondrial dysfunction, impairment of axonal transport, dysfunctional signaling pathways and inflammatory cascades of non-neuronal cells (11, 12).

Few veterinary studies to date have focused on the pathogenesis of MND. The cause of motor neuron death is not attributable to neurofilament accumulation alone. Synaptophysin is a synaptic vescicle-associated glycoprotein expressed by motor neurons. Immunohistochemical analysis of synaptophysin expression on affected Holstein Friesian calves had been performed in order to investigate whether the lower motor neuron degeneration was a result of loss of afferent contacts. The study revealed decreased expression of synaptophysin on affected neurons, suggesting progressive loss of pre-synaptic terminals because of abnormal signaling of target motor neurons rather than primary loss of afferents, and no evidence of apoptotic signals or caspase 1 and 3 activation, which are believed to have a pivotal role in mediating cell death (4, 13). Furthermore, diffuse astrogliosis associated with neuronal degeneration, as seen in the present cases, is a well-described feature of MND in human medicine (12).

Historically, several types of SMA in human medicine have been differentiated according to age at onset and clinical course (SMA I–IV). The first two have their onset in infancy and affected patients never achieve the ability to sit unassisted (Type I, also referred as Werding-Horrman disease) or walk independently (Type II—Dubowitz disease). In SMA type III (also known as Kugerberg-Welander disease), clinical signs become apparent around 1 year of age, with progressive proximal weakness of the legs in particular. SMA type IV, which accounts for <5% of SMA cases, has its onset in adulthood, progresses slowly, and is relatively benign (14, 15). Another type of SMA has been defined as SMA type 0 because of its prenatal onset. Neonates have a history of decreased fetal movements and present with severe weakness, hypotonia, areflexia, and facial diplegia. Cardiac defects and joint contractures are concurrently reported; life expectancy is severely shortened due to respiratory failure (15). Although involvement of brain structures is poorly documented in patients with SMA, neuroimaging of the cortical gray matter revealed supratentorial atrophy in three patients with SMA type 0, whereas the thalamus, basal ganglia, and cerebellum appeared normal and the white matter displayed a normal myelination pattern (16).

Based on the medical history of the calves in this series (recumbency since birth), a diagnosis of SMA type 0 appears plausible, though the lack of respiratory muscle involvement and brain lesions tends to make a diagnosis of SMA type I more likely, as suggested for other bovine breeds.

Most (95%) cases of SMA in human medicine are associated with mutations in the SMN1 exon 7 gene located on chromosome 5q13, with an autosomal recessive mode of inheritance. The SMN1 protein product is ubiquitous in all cells and important in the formation of spliceosomes involved in the processing of pre-mRNA into mRNA (17); however, it is uncertain why motor neurons are particularly vulnerable for this defect (18).

Taking advantage of the remarkable similarities between human and bovine SMA, genetic investigations have been carried out on Braunvieh breed. In 2003, Medugorac and colleagues initially hypothesized that the apoptosis-inhibiting protein BCL2, located on chromosome 24, was the most promising positional candidate gene (19). Fine-mapping of the bovine SMA locus suggested other positional candidates, including its immediate neighbor FVT1, which encodes KDSR, involved in glycosphingolipid metabolism, and the VPA4B gene, involved in other neurodegenerative diseases (20). In 2007, definitive mapping of the functional mutation causing SMA in Braunvieh to the FVT1 gene demonstrated that, despite the clinical and pathological similarities, bovine and human SMA were caused by mutations in completely different genes (8). No mutation of the FVT1 gene in our patients was found on genetic analysis. Nonetheless, the mutation described for the Braunvieh might be merely a linked marker in that specific breed and not the causative mutation of SMA (8). Several other enzymes in the glycosphingolipid pathways are known to be responsible for neurodegenerative disorders in cattle, and the results in the Blonde d'Aquitaine could be due to a difference in the level of linkage disequlibrium between the two breeds, which was responsible for the loss of the relationship between the marker and the real causative mutation of SMA in our samples.

In 4–5% of human patients with clinical signs typical of SMA, no identifiable mutation in the SMN1 gene has been found, though a number of non-5q causative genes associated with SMA have been identified to date (21, 22). Other genetic mutations need to be identified, and naturally occurring animal models provide an important resource. A mutation screen of the bovine and the feline SMA gene in a cohort of 96 human patients with atypical SMA following the identification of genetic mutations associated with SMA in bovine and feline patients (2, 8, 23) failed to find mutations in coding sequences or splice sites in human orthologs, however (24).

Study of the causative gene mutation of SMA in these Blonde D'Aquitaine calves is currently under way. The identification of a novel genetic mutation may potentially improve our understanding of the disease in human medicine.

This is the first report of SMA in Blonde d'Aquitaine calves. Contrary to previous reports for other bovine breeds, genetic analysis of the calves and their relatives excluded the involvement of FVT1 polymorphism in the development of the disease. Identification of the causative genetic mutation in this breed could improve our understanding of SMA.

## Data Availability Statement

All datasets presented in this study are included in the article/ supplementary material.

## Ethics Statement

Ethical review and approval was not required for the animal study because not required for restrospective case report. Written informed consent was obtained from the owners for the participation of their animals in this study.

## Author Contributions

GC helped manage patient from diagnosis through euthanasia, conceived the case report, drafted the article, critically revised the article, and gave final approval of version to be published. CC interpreted the histopathology, critically revised the article, and gave final approval of the version to be published. SC and PS performed the genetic investigation, critically revised the article, and gave final approval of the version to be published. CB and AD'A helped with patient management from diagnosis through euthanasia, conceived the case report, critically revised the article, and gave final approval of the version to be published. All authors contributed to the article and approved the submitted version.

## Conflict of Interest

The authors declare that the research was conducted in the absence of any commercial or financial relationships that could be construed as a potential conflict of interest.

## Abbreviations

SMA: Spinal muscular atrophy
KDSR: 3-ketodihydrosphingosine reductase
MND: motor neuron disease.
